# Potential of Point-of-Care and At-Home Assessment of Immune Status via Rapid Cytokine Detection and Questionnaire-Based Anamnesis

**DOI:** 10.3390/s21154960

**Published:** 2021-07-21

**Authors:** Noor Jamaludeen, Christian Beyer, Ulrike Billing, Katrin Vogel, Monika Brunner-Weinzierl, Myra Spiliopoulou

**Affiliations:** 1 Knowledge Management & Discovery Lab, Otto-von-Guericke University, 39106 Magdeburg, Germany; christian.beyer@ovgu.de (C.B.); myra@ovgu.de (M.S.); 2 Department of Experimental Pediatrics, University Hospital, Otto-von-Guericke University, 39120 Magdeburg, Germany; ulrike.billing@med.ovgu.de (U.B.); katrin.vogel@med.ovgu.de (K.V.); monika.brunner-weinzierl@med.ovgu.de (M.B.-W.)

**Keywords:** rapid cytokine detection, immunoassay, health questionnaire, data mining, smartphone- based signal reader, lateral flow assay, point-of-care, at-home testing, topic modeling

## Abstract

Monitoring the immune system’s status has emerged as an urgent demand in critical health conditions. The circulating cytokine levels in the blood reflect a thorough insight into the immune system status. Indeed, measuring one cytokine may deliver more information equivalent to detecting multiple diseases at a time. However, if the reported cytokine levels are interpreted with considering lifestyle and any comorbid health conditions for the individual, this will promote a more precise assessment of the immune status. Therefore, this study addresses the most recent advanced assays that deliver rapid, accurate measuring of the cytokine levels in human blood, focusing on add-on potentials for point-of-care (PoC) or personal at-home usage, and investigates existing health questionnaires as supportive assessment tools that collect all necessary information for the concrete analysis of the measured cytokine levels. We introduced a ten-dimensional featuring of cytokine measurement assays. We found 15 rapid cytokine assays with assay time less than 1 h; some could operate on unprocessed blood samples, while others are mature commercial products available in the market. In addition, we retrieved several health questionnaires that addressed various health conditions such as chronic diseases and psychological issues. Then, we present a machine learning-based solution to determine what makes the immune system fit. To this end, we discuss how to employ topic modeling for deriving the definition of immune fitness automatically from literature. Finally, we propose a prototype model to assess the fitness of the immune system through leveraging the derived definition of the immune fitness, the cytokine measurements delivered by a rapid PoC immunoassay, and the complementary information collected by the health questionnaire about other health factors. In conclusion, we discovered various advanced rapid cytokine detection technologies that are promising candidates for point-of-care or at-home usage; if paired with a health status questionnaire, the assessment of the immune system status becomes solid and we demonstrated potentials for promoting the assessment tool with data mining techniques.

## 1. Introduction

The function of the human immune system is to protect the body from a variety of diseases [1]. Cytokines are protein substances that regulate the immune response in health and disease [2]. The dysregulations in the cytokines levels play a major role in the pathophysiology of a range of autoimmune diseases, infectious diseases, and allograft rejection (e.g., IL-1, IL-4, IL-6, IL-10, IL-12, TNF-α, and IFN-α, -β, -γ) [3]. Cytokines reveal a broad spectrum of health issues. For example, Interleukin-6 (IL-6) is a multifunctional cytokine, and high bloodstream levels of it have been associated with severe inflammatory diseases [4], such as dengue fever, sepsis, various cancers, and visceral leishmaniasis (VL) [5]. Therefore, instead of testing the patient for four diseases, running one cytokine test would be sufficient to detect any health issue even for asymptomatic diseases. However, quantitation of cytokine levels alone do not reflect the status of the immune system precisely. Further information on the patient’s health condition and lifestyle is required, as well. For example, environmental influences make the variability of cytokine ranges very large among individuals. Therefore, numerous confounding factors affect the immune system and must be taken into account when assessing the immune system response [6]. For this particular purpose, many health questionnaires were established to juxtapose the clinical assessment of the case with the laboratory tests.

Quantitation of cytokine levels is sophisticated time-consuming task. Enzyme-linked Immunosorbent Assay (ELISA) is a commonly used assay for the precise quantitation of cytokines. ELISAs consume from 3 to 8 h, are conducted in specialized laboratories, and require advanced equipment. In critical medical conditions, it is crucial to have rapid, high-thorough frequent measurements of the cytokine levels where using ELISA is not a wise practice. As in Sepsis, which is one of the most common causes of death for hospitalized patients, Interleukin (IL)-6 reaches peak levels rapidly within 2 h after exposure to an infectious stimulus [7].

People with chronic diseases have to go through regular, time-wasting, painful, due to the multiple blood withdrawals, and costly check ups. Early detection in critical health conditions not only saves lives but also saves time, as well as medical resources.

Therefore, an urgent demand has emerged to develop assays capable of delivering rapid measurements of the cytokine levels from small volume of samples. Point of care (PoC) assays have recently witnessed impressive progression and paved the way to conduct a rapid assessment of the immune system in clinics through cytokine detection or even push the limits further towards self-assessment of the immune status [8] at home.

Former studies have already addressed recent advances in cytokine detection but did not stress on the rapidness of the cytokine measurements. Liu et al., in their review [9], discussed the most recent robust advances achieved in cytokine detection up to 2016. We collect recent advances from 2015 to 2020 with a focus on the *rapid* total assay time. Another study focused on optical and electrochemical-based technologies for measuring IL- 6 [10]. In contrast, our work is not limited to one particular cytokine. We are interested in all cytokines that may play a major role in the immune system. Our study mainly focuses on the rapidness of the method, which can be achieved through short assay time and simultaneous quantification of cytokines (i.e., multiplex immunoassay).

Throughout this work, we aim to answer the following research questions on rapid assessment of immune status through cytokine detection:

- $RQ_{1}$: What recent technologies are developed for the rapid determination of cytokines?
- $RQ_{2}$: Which of these rapid technologies can measure multiple cytokines simultaneously?
- $RQ_{3}$: Which of these rapid technologies are suitable for Point-of-Care (PoC) testing?
- $RQ_{4}$: Which of these rapid technologies are suitable for At-Home testing?
- $RQ_{5}$: What health questionnaires exist for health status assessment?
- $RQ_{6}$: How can we link the collected health questionnaires with the identified rapid technologies?
- $RQ_{7}$: How can we employ machine learning strategies to automatically derive the definition of the immune system’s fitness from literature?
- $RQ_{8}$: How can we build an integrated system that assesses the person’s immune system fitness?

It is stressed that we distinguish between testing at home (cf. $RQ_{4}$), eventually by the patients themselves (self-testing), and testing at the point of care ($RQ_{3}$), which we perceive as a premises with appropriate equipment for the acquisition, maintenance, and processing of samples.

**Figure 1:**
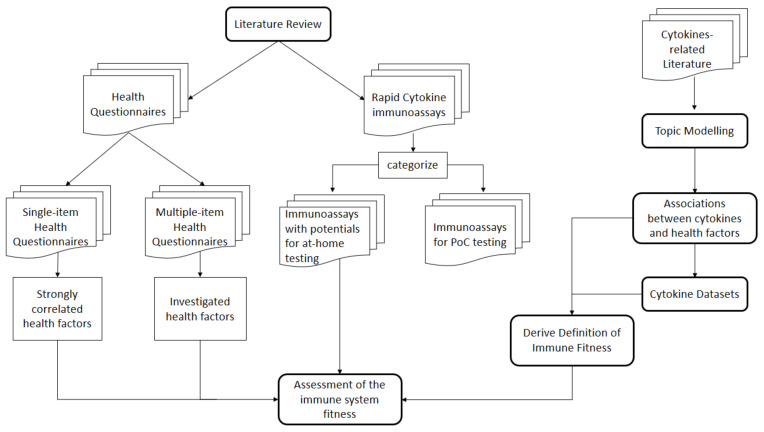
An abstract description of this work’s objectives.

To answer $RQ_{1}$, $RQ_{2}$, and $RQ_{5}$, we follow the systematic literature review (SLR) approach to retrieve relevant studies. Then, we answer $RQ_{3}$ and $RQ_{4}$ by defining ten criteria to investigate whether the identified rapid cytokine detection assays are appropriate for PoC or represent promising candidate for at-home usage. Similarly, we compare the retrieved health questionnaires on the basis of three defined criteria. $RQ_{6}$ is addressed through establishing the link between the health aspects investigated by the health questionnaires and the cytokines identified by the rapid cytokine detection assays. We rely on text mining, more specifically on topic modeling, to establish a concrete definition of the fitness of the immune system to answer $RQ_{7}$. Finally, we present a general description of an integrated health assessment system as an answer to $RQ_{8}$. This system leverages the derived definition of the fitness of the immune system, the cytokine measurements delivered by the rapid cytokine immunoassay, and the complementary information collected by the health questionnaire to assess the fitness of the immune system. Figure 1 summarizes the main objectives of this work.

In Section 2, we describe the literature review procedure, discuss how we plan to (1) identify the requirements for PoC and at-home usage, (2) and the method we follow to pair the identified health questionnaires with the rapid detection methods. Then, we list all the rapid detection assays and the health questionnaires resulted from the literature retrieval in Section 3. Afterward, we study the potential of the retrieved detection assays for PoC and at-home usage and establish the link between the retrieved health questionnaires with the identified rapid detect assays in Section 4. Section 5 connects the health aspects addressed by the collected health questionnaires with the cytokines measured by the retrieved rapid immunoassays. In Section 6, we present a data mining-based solutions to derive the definition of the fitness of the immune system, model the associations between cytokines and other health factors, and introduce an integrated assessment tool of the person’s immune system. Finally, we summarize our work in Section 7.

## 2. Materials and Methods

This section describes the methodology we follow to answer our research questions. First, we follow the systematic literature review (SLR) approach [11]. Guided by the formed research questions, we develop relevant keywords for querying a database of academic papers to retrieve relevant literature in Section 2.1. Afterward, we apply inclusion and exclusion criteria to the retrieved studies in order to filter out irrelevant ones in Section 2.2; then, we divide all relevant studies into three main categories based on the utilized signal detection technique. We define four criteria, demonstrated in Table 1, to investigate whether the rapid cytokine detection assay is appropriate for PoC or represents a promising candidate for at-home usage in Section 2.4. Similarly, in Section 2.3, we define three criteria, which we use to compare between the retained health questionnaires. Finally, we present our method on how to establish the link between the health questionnaires and the identified cytokine detection assays in Section 2.5.

**Figure 2:**
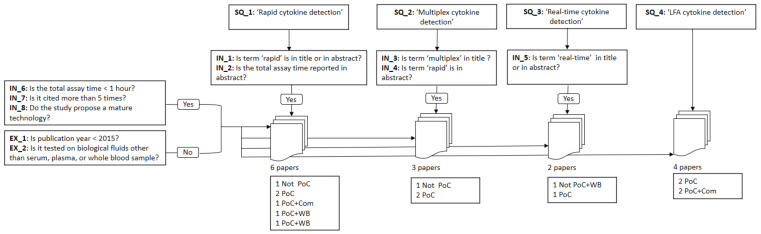
An abstract description of literature retrieval of rapid cytokine detection methods. SQ = search query, IN = inclusion criteria, EX = exclusion criteria, PoC = point-of-care, Com = commercial product in market, WB = whole blood.

**Figure 3:**
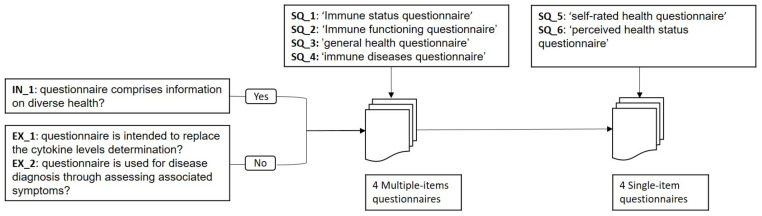
An abstract description of literature retrieval of health questionnaires. SQ = search query, IN = inclusion criteria, EX = exclusion criteria.

The steps of the literature retrieval and selection are described briefly in Figure 2 for the rapid cytokine detection methods and in Figure 3 for the health questionnaires.

### 2.1. Literature Retrieval Methodology

To answer the previous research questions, we follow the systematic literature review (SLR) approach [11] to answer $RQ_{1}$, $RQ_{2}$, and $RQ_{5}$. We form several search queries and posit them to the Google search engine.

The assessment of the immune system fitness necessitates *rapid* cytokine detection, especially in critical health conditions. Therefore, we concentrate our literature retrieval on *rapid* immunoassays or immunoassays capable of delivering *real-time* measurements. In addition, a short total assay time can be achieved through the simultaneous measuring of multiple cytokines. Therefore, we are also interested in *rapid multiplex* cytokine detection that delivers a more accurate assessment of the immune system through measuring multiple cytokines at a time. One well-known family associated with the rapid methods is the *LFAs* due to their short total assay time.

The search queries consist of the following terms that define desirable properties of the targeted immunoassays for the cytokine detection:

- ‘rapid’: an immunoassay must deliver results faster than standard ELISA.
- ‘multiplex’: an immunoassay must measure rapidly multiple cytokines, which is necessary to evaluate the overall immune system status.
- ‘real-time’: immunoassays with superior feature to the rapid detection is the real-time continuous monitoring of the cytokine detection.
- ‘lateral flow assay’: LFAs are rapid tools known for their ease of application.

Using the previous defined terms, we built three search queries:

- SQ_1: “immunoassay for the rapid cytokine detection”,
- SQ_2: “multiplexed immunoassay for the rapid cytokine detection”,
- SQ_3: “immunoassay for real-time cytokine detection”, and
- SQ_4: “LFAs cytokine detection”.

In this study, we emphasize the importance of pairing the cytokine levels determination along with information on the immune status represented by health conditions, lifestyle, physical ability, and immune functioning status by conducting a literature review of various health questionnaires that collect the desired information to evaluate the measured cytokine levels reliably. We elaborate the argument to demonstrate the reasons behind considering the health questionnaire as a crucial component in the design of any powerful immune status assessment platform in Section 3.4. For the retrieval of health questionnaires, we defined the following terms:

- ‘Immune status/functioning’: the questionnaire must aim at evaluating the immune system.
- ‘general health’: the questionnaire must consider several health aspects for the general assessment.
- ‘perceived health stats/self-rated health’: This type of questionnaires comprise a single item which asks: “How would you rate your health status?”
- ‘immune disease questionnaire’: This questionnaire includes items that collects information on immune diseases.

Then, we use them to create the following search queries:

- SQ_1: “Immune status questionnaire”,
- SQ_2: “Immune functioning questionnaire”,
- SQ_3: “general health questionnaire”,
- SQ_4: “self-rated health questionnaire”,
- SQ_5: “perceived health status questionnaire”, and
- SQ_6: “immune diseases questionnaire”.

We performed the search in August 2020 on Google Scholar (https://scholar.google.com/, accessed on 12 August 2020) because it is a well-known electronic literature database, as it encompasses all relevant databases, such as ACM Digital Library and IEEE Xplore, and also allows searching within the full text of a paper.

### 2.2. Literature Selection Methodology

Our inclusion criteria for the first two research questions are as follows:

- For studies retrieved with the search query “immunoassay for the rapid cytokine detection”,
  – IN_1: the study must include the term ‘rapid’ in the title or in the abstract.
  – IN_2: and must report the total assay time in the abstract as an indicator of how important this feature is for the immunoassay.
- For studies retrieved with the search query “multiplexed immunoassay for the rapid cytokine detection”,
  – IN_3: the study must include the term ‘multiplex’ in the title.
  – IN_4: and the term ‘rapid’ in the abstract.
- For studies retrieved with the search query “immunoassay for real-time cytokine detection”,
  – IN_5: the study must include the term ‘real-time’ in the title.
- In addition to four inclusion criteria that must be fulfilled by all the papers retained.
  – IN_6: The total assay time should be less than 1 hour.
  – IN_7: The study should be cited at least 5 times.
  – IN_8: The study must propose a mature technology. This was assessed by reading the abstract of the study.

The exclusion criteria for $RQ_{1}$ and $RQ_{2}$ are:

- EX_1: Studies older than 2015 are not included. This review gathers most recent advanced immunoassays for the rapid cytokine detection.
- EX_2: Immunoassays that were tested on biological fluids other than serum, plasma, or whole blood sample, such as amniotic fluid are also excluded.

For $RQ_{5}$, we retrieve relevant articles on questionnaires. Retrieving relevant questionnaires is a more complicated task than finding relevant rapid immunoassays because, in many studies, the title does not express well the purpose of the questionnaire. Some studies present the questionnaire as an alternative tool to measuring cytokines circulating in blood. For example, the pro-inflammatory cytokine levels are correlated with the symptoms severity of spinal disorders questionnaire [12], the Hip disability and osteoarthritis questionnaires [13], self-rated health questionnaire [14], and the sickness behavior questionnaire [15], whereas others present questionnaires as tools to support the diagnostic decision for one particular disease [16]. This survey discusses questionnaires as general health assessment tools that provide additional information, independent from results obtained from measuring any bio-markers. As a result, we define the following inclusion and exclusion criteria to answer the fifth research question:

- IN_1: the questionnaire must comprise information on as diverse health aspects as possible.

And we exclude questionnaires that:

- EX_1: are intended to replace the cytokine levels determination.
- EX_2: are used for disease diagnosis through assessing associated symptoms.

After applying the inclusion and the exclusion criteria, we end up with many studies that propose solutions based on similar technologies. We compare between these studies and select the one with either the higher number of citations or is identified as the state-of-the-art as the representative study of this technology.

### 2.3. Feature Selection for the Identified Questionnaires

We identify multiple-item questionnaires [17,18,19,20] and single-item questionnaires [21,22,23,24]. For these juxtaposition of the questionnaires, we consider the following set of features: (1) *‘major health factors’*correlated with the questionnaire (these health factors are important to identify in next steps the cytokines that must be measured by the rapid cytokine assay to assess the immune system), (2) *‘scale of the score’* on which the correlation with the investigated health factors are discovered, and (3) *‘number of questionnaire items’* to assess the effort required from the patient/user to fill the questionnaire.

### 2.4. Categorization and Feature Selection for the Identified Detection Methods

We categorize the methods of the retained papers on cytokine detection into three different categories [25] based on the signal detection approach they utilize: (1) Optical signal detection [26,27,28,29,30], (2) Electrochemical signal detection [31,32,33,34,35], and (3) Colorimetric signal detection [5,36,37], LFA (Milenia Biotec) and LFA (Antagen hIL-8 XpressCard).

Our study stresses the rapidness of cytokine detection through collecting methods that are rapid and measure multiple cytokines simultaneously. However, it is not necessary that all the rapid immunoassays be appropriate for PoC or at-home testing. Therefore, to assess the appropriateness of the retrieved methods, we compare them on the following features:

- *Sample:* sample volume (mostly μL) and type (e.g., serum),
- *Cytokines:* set of simultaneously detected cytokines,
- *Dynamic Range (DR)*,
- *Limit of Detection (LoD)*,
- *Validity of measurements*: it is used to assess the reliability of the method,
- *Assay time*,
- *Signal reader:* signal processing method, and reading equipment, e.g., scanner, special type of camera, smartphone (camera),
- *Specific disease*, marking cases where the technique is intended for specific disease(s) only
- *Dimensions* of the different parts of the immunoassay, and
- *Cost* of fabrication and materials used.

WHO stresses that the technology used at PoC has to satisfy the ASSURED requirements, which are (affordable, sensitive, specific, user-friendly, rapid and robust, equipment-free, and delivered). These criteria can be mapped to our criteria as follows: ’affordability’ can be assessed by the *cost*; ‘user-friendly’ can be conjectured from the *sample size*, *sample type*, and *dimensions*; the PoC should be of a small size *dimensions* of the device, require a tiny *sample size*, and preferably can operate on unprocessed blood (*sample type*); and ‘equipment-free’, in that there is no need for a *signal reader*. A ‘rapid’ PoC has a short *assay time*. *Limit of Detection (LoD)* and *Dynamic Range (DR)* [38] reflects the sensitivity of the PoC. Most of the studies collected throughout our work are not interested in ‘specificity’ as an evaluation metric brcause they are not concerned with the diagnosis of a disease but, rather, with the accurate measurement of the cytokine levels in the blood to assess the immune system fitness. Therefore, they replaced specificity with other validation metrics used to assess the accuracy of the immunoassay. The accuracy of most of the assays was validated with the linear correlation between the produced measurements and measurements generated by the gold standard ELISA. Some studies reported the coefficient of variation CV to express the precision and repeatability of an assay and the recovery [39].

We verify the appropriateness of these features for the assessment of PoC versus at-home testing by juxtaposing them with features deemed important in recent reviews on immunoassay readers for at-home testing. To this purpose, we acquire reviews with following 4-step subprocess.

Step 1: Preliminary literature collection and inspection

To assess the potential of technologies for at-home assessment, we first collect literature reported from 2017 onwards, using the keywords ‘point of care’, ‘device’, and ‘whole blood’ on scholar.google.org. The last keyword is used because many of the identified technologies are based on whole blood samples. We exclude articles that referred to a specific condition, like sepsis, HIV, or cancer. A cursory inspection of some of the retained articles indicates a focus on advances appropriate for self-testing and, thus, for at-home use [40,41,42].

Step 2: Collection of recent literature

Moved by the insight that many of the articles refer to a particular condition, we perform a backward, year-based search on scholar.google.com until and including 2017, with the keywords ‘self-testing’, ‘device’, and ‘immunoassay’. We exclude articles on a specific condition (e.g., hepatitis) or a specific substance (e.g., cortisol). We obtain the following hits:

- Three hits from 2020 onwards [42,43,44]. One hit [42] has already been identified in the preliminary literature collection), another article [43] appears twice, once under the name of the author (Andryukov) and once under the forename of the author (Boris), and another hit [44] is excluded because it does not refer to self-testing.
- One hit in 2019 [45].
- Three hits in 2018 [40,46,47]. One of them [40] has already been identified in the preliminary literature collection, and another [47] is excluded because it does not contain any publication data.
- Five hits in 2017 [48,49,50,51,52].

Step 3: Literature selection

We merge these hits with those of the preliminary search. From these, we retain those that review or otherwise discuss recent advances, thus excluding articles that concentrate on a specific technological solution. Eleven articles are finally retained [40,41,42,43,45,46,50,51,52,53,54], ordered alphabetically by name of the first author.

None of these articles refers to the measurement of cytokines, although two of them [43,50], mention the potential of the discussed technologies for cytometry. Hence, there is no overlap between these reviews and ours. We use these reviews exclusively to identify characteristics that promote self-testing.

Step 4: Extraction of characteristics that indicate potential for self-testing

We inspect the articles finally retained at Step 3 of the literature selection to extract features that seem characteristic of technologies appropriate for self-testing and, thus, for at-home measurements. Since our focus is on cytokines, we concentrate on features associated with optical reading, i.e., ignored features intended to capture audio signal, vibration, movement, etc.

We find that smartphone cameras/lens, possibly with a mechanical adapter attached to it, are seen as appropriate for image processing [41,50,54] and readings from paper (test pads, test strips) [40]. Zarei points to the potential of smartphones for all of ‘colorimetric, fluorescence, luminescence, and electrochemical detections’ [52]. Some readers can be attached to a smartphone [53]. Other readers can be linked to wearables [54], while others point to ‘sample handling platforms, recognition elements and sensing methods’ that can be attached to a smartphone or wearable [42]. Hence, we consider cytokine reading devices that can be attached to a smartphone as appropriate for at-home usage.

In the discussion of ‘six decades of lateral flow immunoassay’, Andryukov points to several advantages of lateral flow immunoassay (LFIA) methods (cf. Table 2 of (Andryukov et al., 2020) [43]), including ‘cheap, rapid, affordable’, ‘small size of analyzed sample’, ‘long shelf-life of test systems’, and ‘do not require special temperature conditions for storage’. The sample size and the temperature are also important features [50,54]. Nayak et al. consider blood acquisition (also for the acquisition of whole blood) through finger prick, and they stress humidity conditions as a further feature of importance [50].

From these, we derive following criteria of relevance:support for smartphone/wearable, including readers that can be attached to a smartphone/wearable; price (lower is better); assessment speed (high/rapid is better); size of analyzed sample (smaller is better); temperature (requiring no special temperature is better); and shelf-life of test system (longer is better). Since price of the technologies we study cannot be always assessed, and since the same amount of money can be perceived as high, resp. low, in different countries, we exclude this feature from the comparison between the immunoassays and just report it when available.

## 2.5. Combination of Sources and Features for Cytokine-Based Immune System Status Assessment

We use the features identified for detection techniques and for questionnaires to come to a combination of sources + features appropriate for PoC testing and for at-home cytokine assessment. The health questionnaires investigate a variety of major health factors. These major factors reflect the status of the immune system represented by the cytokine levels. Therefore, we conduct a literature review to discover the cytokines that affect each of these major health factors. Then, we couple each of the health questionnaires to the immunoassay that measure the corresponding cytokines. Since we focus our work on the collected immunoassays, cytokines that are not measured by any of the retrieved immunoassays are excluded.

## 3. Results

Our search results in a list of 15 rapid cytokine assays adhering to three signal detection techniques: optical detection (group O), electrochemical detection (group E), and colorimetric detection (group C). We categorize them with respect to the features of Section 2.4 and summarize them in Table 2 and Table 3 (group O), Table 4 and Table 5 (group E), and Table 6 (group C). The last column refers to the *Usage*, whereupon we distinguish between studies reported the possibility of Point-of-Care (PoC) and at-home testing and those that do not. Assays reported in studies as commercial medical devices available in market are marked additionally with a (${}^{☆}$).

### 3.1. Optical Detection Techniques

Optical detection has been witnessing an increasing involvement in the design of point-of-care devices. This technique relies on the sensitive detection of photon emission from dyes and molecules excitable by light [25]. Five papers that built their immunoassay on optical detection are identified with the previously described research procedure and are depicted in Table 2.

Three out of these five assays utilized mainly the Localized Surface Plasmon Resonance (LSPR) technology [26,27,29]. One biosensing device (LSPR-AuNRs) was fabricated primarily on this technology [26] using antibody conjugation of gold nanorods (AuNRs). Another device (LSPR-FACSNPs) incorporated the LSPR dark-field imaging technique with a magnet patterned Fe${}_{3}$O${}_{4}$/Au core–shell nanoparticles (FACSNPs) sensing array [27], whereas the (LSPR-MoS2)-based device is an LSPR biosensor integrated with few-layer molybdenum disulfide (MoS2) photoconductive component [29]. One of the two other studies followed multiplexed femtomolar quantitation in a fluoropolymer microcapillary film (MCF) [30], and the other proposed a photonic lab-on-a-chip design for cytokine detection (PhLoC) [28].

As can be seen in the 2nd column, *Sample*, of Table 2, the first three immunoassays of this group [26,27,28] operated on very small samples of 1 to 5 μL. The first two immunoassays consumed tiny samples 1 μL of blood serum to measure 4–6 cytokines simultaneously (cf. column *Cytokines*), while the (MCF) immunoassay [30] demanded a larger sample volume of 150 μL to capture multiple cytokines. The *Assay time* (7th column) varied from 40 min for the measurement of 6 cytokines [26] down to real time for the 4 cytokines measured by the (LSPR-FACSNPs) immunoassay [27] and varied from 10 to 20 min for the other three methods. As can be seen in the column *Specific disease*, the (LSPR-AuNRs)-based immunoassay at the top-row was intended for the detection of inflammatory responses after cardio-pulmonary bypass (CBP) surgery, while the other immunoassays of this group were not specialized for some specific condition, i.e., they were appropriate for disease-independent usage.

From the 4th and 5th columns in Table 2, we see that the three (LSPR)-based methods showed superior performance to other methods with respect to the large *dynamic ranges* and low *LoD*. The(LSPR-AuNRs)-based [26] and the (LSPR-MoS2) [29] immunoassays reported the largest *dynamic range* and the lowest *LoD* for IL-6, IL-2, and TNF-α and for IL-1β, respectively, among all the other methods.

All the studies in this category validated their measurements of cytokine levels by comparing their results with the gold standard conventional ELISA. The (LSPR-AuNRs)-based immunoassay [26] reported a high correlation with the multiplex ELISA with $R^{2}$ = 0.9726, and a recovery within an acceptable range. The (LSPR-FACSNPs) immunoassay [27] also reported a high correlation, with multiplex ELISA with $R^{2}$ = 0.9252. The (MCF) immunoassay [30] had the coefficient of variations of all measurements within an acceptable range <10%. The (LSPR-MoS2) [29] and the (PhLoC) immunoassays [28] just stated that their results were consistent with the conventional ELISA and, in some cases, were even better concerning the *LoD*.

The *Signal reader* techniques (cf. 8th column of Table 2) varied substantially. To quantify the scattering light intensity obtained by the (LSPR)-based immunoassays, dark-field imaging optics were used by the (LSPR-AuNRs)-based [26] and the (LSPR-FACSNPs) [27] immunoassays. The technique of Castanheira et al. for singleplex and multiplex cytokine detection [30] (last row of Table 2) needed an HP ScanJet G4050 Scanner similar in complexity to the signal readers used by all the immunoassays in this group. In contrast, the (LSPR-MoS2)-based immunoassay [29] (2nd row from bottom of the table) did not require a complex signal reader because it utilized an ultra-sensitive MoS2 photodetector. The (PhloC) immunoassay [28] (the 3rd one from bottom to top) used a microscope with a unit filter to detect the UV-vis light transmitted through the measuring chamber; moreover, the structure of this assay allowed for ease multiplexing.

Further information about the design of these assays can be found in Table 3. The column *Dimensions* reflects the size and the different parts of the assay. We notice that all the assays comprised multiple parts that were integrated all together on one platform. The largest dimension was 2 cm, and the dimensions ranged from 25 μ to 2 cm. The place of *reaction* is also included in Table 3. None of these assays reported the cost of fabrication or materials used.

Concerning Usage at PoC or at-home (cf. last column of Table 2), all but the first of these immunoassays reported that they were appropriate for PoC testing. And, none of them reported the potential for at-home testing. However, the small or even tiny sample volumes demanded for the detection of one or more cytokines and the short assay time made them particularly attractive for At-Home usage. However, the additional equipment needed for the readouts made it challenging to deploy them for at-home testing.

### 3.2. Electrochemical Detection Techniques

Electrochemical (EC) biosensors are principled on the transformation of biochemical information which would be the cytokine concentrations into an analytically interpretable signal, such as current or voltage [55]. We identify five studies that were based on electrochemical detection: (1) a biosensor based on aptamer technology to release a drug upon the detection of target cytokine [31]; (2) a multiplexed electrochemical microfluidic immunoarray with eight 32-sensor electrochemical arrays connected with a miniaturized 8-port manifold [32]; (3) an immunoassay with a sequentially multiplexed amperometry on a single-chip potentiostat [33]; (4) a testing platform built on Proxim Technology (San Jose, CA, USA) [34]; and (5) a hybrid magneto-electrochemical miniaturized sensor [35].

Immunoassays in this group exhibited large variances in the required sample volumes. The sample volumes displayed in the 2nd column in Table 4 varied from 5 μL to significantly larger volumes of 150 μL. The 8-port manifold microfluidic biosensor [32] consumed the smallest sample volume 5 μL, whereas two electrochemical-based PoC immunoassays that were advanced mature devices are the PoC-Proxim device [34] which is available in the market and the hybrid magneto-based biosensor [35] (cf. in the last two rows in Table 4), which required relatively larger sample volumes of 100 μL. The largest sample volume 150 μL was consumed by the sequentially-multiplexed biosensor [33]. All previously listed immunoassays delivered measures in vitro. One immunoassay in this family that could deliver measurements in vivo was the aptamer-based biosensor [31] (cf. 1st row in Table 4). It is important to mention that the aptamer-based biosensor [31] was tested on rats.

The number of cytokines that could be measured simultaneously by each immunoassay can be found in the 3rd column. The aptamer-based [31], the one commercial (PoC-Proxim) [34], and the hybrid magneto-based [35] biosensors could only measure one single cytokine per test, as can be seen in the 3rd column in Table 4. The 8-port manifold microfluidic [32] and the sequentially-multiplexed [33] biosensors were customized to tackle this limitation and could simultaneously measure two cytokines in addition to two proteins and one cytokine in addition to one more protein, respectively. This indicates their high sensitivity in detecting cytokines in complex samples.

The 8-port manifold microfluidic [32] and the PoC-Proxim [34] biosensors for IL-6 quantification had relatively wide dynamic ranges compared to the optical (LSPR-AuNRs)- based immunoassay [26], as shown in the 4th column in Table 4. The lowest *LoD* for IL-6 in this category was reported by the commercial PoC-Proxim device (Proxim, CA, USA) [34] (cf. 4th row and 5th column in Table 4).

The 8-port manifold microfluidic [32] and the PoC-Proxim [34] biosensors validated their measurements with the comparison to the conventional singleplex ELISA and reported R${}^{2}$ > 0.96, as can be seen in the 6th column. The PoC-Proxim biosensor [34] proved the precision and the accuracy of the standard curve and controls with acceptable coefficients of variations CV within 14% (cf. the 4th row and the 6th column in Table 4). The hybrid magneto-based biosensor [35] was tested on 62 clinical samples from septic and non-septic patients and reported sensitivity = 91.3% and specificity = 82.4%.

As shown in the 7th column in Table 4, the aptamer-based biosensor [31] delivered real-time measurements in vivo compared to a total assay time of 1 h in vitro. The shortest assay time (20 min) was achieved by the commercial PoC device (Proxim, CA, USA) [34]. The assay time required by the remaining biosensors [32,33,35] ranged from 40 min to ≤1 h.

The PoC-Proxim [34] and the hybrid magneto-based [35] biosensors relied on smartphones for the signal reading, whereas all the remaining immunoassays in this group demanded a potentiostat, as in the 8-port manifold microfluidic [32] and the sequentially-multiplexed [33] biosensors, with an additional UV–Vis spectrophotometer, as in the aptamer-based biosensor [31].

Regarding the diseases targeted by these immunoassays (cf. the 9th column in Table 4), the aptamer-based [31] and the sequentially-multiplexed [33] biosensors were employed to detect inflammation. The PoC-Proxim [34] and the hybrid magneto-based [35] biosensors were developed to detect Sepsis. The 8-port manifold microfluidic biosensor [32] measured two cytokines for detecting prostate cancer.

Only three immunoassays [33,34,35] in this group were presented as convenient for PoC usage, as demonstrated in the last column of Table 4. Like the optical detection immunoassays, none of the listed assays in this group reported potential for at-home testing.

Further information about the structure of these assays can be found in Table 5. From the column *Dimensions*, we notice that the dimensions of the immunoassays in this group were relatively larger compared to the optical-based immunoassays. This has been already reflected by the larger sample sizes required by the immunoassays in this group. Dimensions ranged from mm to inch. Details on the place of *reaction* is also included in Table 5. Unlike the optical-based immunoassays, some of these immunoassays reported the cost of fabrication and materials used, as they were fabricated to serve as mature commercial products.

Similar to the optical-based techniques, electrochemical immunoassays demonstrated prominent adaptability, as well as great potential to determine the concentration of multiple analytes, within a complex sample in a short time. However, they did not require the complex instruments the optical-based methods needed. The miniaturized structure of electrochemical chips promoted the development of low-cost multiplexed electrochemical microfluidic-based immunoassays proper for PoC practice. In vivo, biosensing was achievable by electrochemical detection for a single cytokine [31]. In general, most of the electrochemical immunoassays asked for sample volumes larger than the optical immunoassays. A multisensor platform based on electrochemical detection required the smallest sample size of 5 μL [32], compared to the smallest sample volume of 1 μL consumed by optical-based immunoassays [26,27].

### 3.3. Colorimetric Detection Techniques

Colorimetric detection technique essentially depends on signal detection by the naked eye, which makes it an ideal instrument for PoC and at-home testing. Compared to the optical and electrochemical techniques, colorimetric techniques do not rely on any expensive advanced signal detection, such as microscopes, a supply of electricity, electrodes, etc. We find a plasmonic mobile biosensor [36] that belongs to this technique was capable of measuring cytokine from unprocessed whole blood samples in vitro. It utilized a smartphone as a signal detector and not the naked eye. The detection process took place on a filter paper (2.5 × 10 cm).

In addition, lateral flow immunoassays (LFAs), known for their simplicity and ease of application, are good examples of assays that rely on this detection technique. We list three LFAs that measured the most inspected pro-inflammatory cytokine IL-6 [5,37] and commercial LFA (https://www.milenia-biotec.com/en/product/il6/ (accessed on 12 August 2020)) (Milenia Biotec), and a commercial LFA that measured IL-8 (https://antagen.net/rapid-human-interleukin-8-xpresscard-atg-il-8/ (accessed on 12 August 2020)) (Antagen hIL-8 XpressCard), which is also an inflammation related cytokine. We have observed that three different elements were conjugated with the cytokine antibodies by the LFAs for the cytokines detection: (1) the LFA-AuNPs [5] and LFA (Milenia Biotec) utilized gold nanoparticles (AuNPs), (2) IL-8 (Antagen hIL-8 XpressCard) used colloidal gold, and (3) the LFA-CdTe QDs [37] conjugated a complex of staphylococcal protein A and fluorescent cadmium telluride quantum dots (SPA-QDs) with the cytokine antibodies.

Similar to the electrochemical group, immunoassays in this group also showed a large variance in the sample size consumed. In the 2nd column from Table 6, the LFA-AuNPs immunoassay [5], the LFA-CdTe QDs immunoassay [37], Antagen hIL-8 XpressCard test and LFA (Milena Biotic) test required samples with volumes ranged from 50 μL to 150 μL. In contrast, the plasmonic-based mobile biosensor [36] consumed relatively tiny sample volumes equals 2.5 μL comparable to the smallest sample volume (1 μL) reported by the LSPR-AuNRs [26] and the LSPR-FACSNPs [27] immunoassays. Not only this, but the plasmonic-based mobile biosensor [36] was also the only immunoassay in this group that could operate on unprocessed blood samples, which made it more appealing to at-home usage.

Contrary to the electrochemical and optical groups, none of the immunoassays in this group could detect multiple cytokines simultaneously, as shown in the 3rd column in Table 6.

Antagen hIL-8 XpressCard test needed the least amount of time (5 min, cf. 7th column in Table 6) to deliver a measurement, while 17 min were required by the plasmonic-based mobile biosensor [36] to generate the results. A total assay time of 20 min was required by the LFA-AuNPs immunoassay [5] and LFA (Milena Biotic) test, whereas the LFA-CdTe QDs immunoassay [37] required the longest total assay time of 30 min.

In this group, we find that the plasmonic-based mobile biosensor [36], for IL-6 detection, reported the lowest *LoD* of 0.1 pg/mL with 99% confidence across all the assays. The LFA (Milena Biotic) test showed a comparable *dynamic range* to the commercial PoC-Proxim device [34] and the LSPR-AuNRs [26].

As can be seen in the *validity of measurements* column and the 2nd row in Table 6, no statistical difference was found between measurements produced by the LFA-AuNPs immunoassay [5] and cytometric bead array (CBA). A very high correlation R${}^{2}$ = 0.9994 was reported between the LFA-CdTe QDs immunoassay [37] and singleplex ELISA (cf. the 3rd row and the 6th column in Table 6), in addition to the small coefficients of variations (CV < 7%) that reflected the stability of the method.

The signal detection in this group did not require complex signal readers, as in the other two groups. Qualitative measurements were feasible through the naked eye for all the LFAs. However, the LFA-CdTe QDs immunoassay [37], the LFA-AuNPs immunoassay [5], and the LFA (Milena Biotic) test could conduct quantitative readings using Canon T61camera with 18–55 lens, portable fluorescence reader (PorFloR${}^{TM}$), and densitometry (PicoScan) reader/chip reading card, respectively. The recent revolutionary progress witnessed in the smartphone industry has presented smartphones as portable, relatively low cost, and with strong cameras, as well as integrated artificial intelligence-based methods for image processing, thus, encouraging the integration of smartphones as powerful signal readers, as in the plasmonic-based mobile biosensor [36].

All immunoassays, except for the plasmonic-based mobile biosensor [36], were presented for PoC usage. Two of the PoC tests, the Antagen hIL-8 XpressCard test and the LFA (Milena Biotic) test, were commercial products.

IL-6 was measured by the plasmonic-based mobile biosensor [36] and the LFA-AuNPs immunoassay [5] to detect sepsis and visceral leishmania, respectively. In addition to IL-6, the LFA-CdTe QDs immunoassay [37] measured the protein CRP to detect orthopedic implant associated infections. Antagen hIL-8 XpressCard test was utilized to detect primary and secondary infections.

Generally, we notice that most of the above listed LFAs had identical total assay time ∼20 min. However, Antagen hIL-8 XpressCard reported the shortest assay time of 5 min. Among the four LFAs, the LFA that reported the smallest sample volume (50 μL) utilized SPA-QDs complex for the detection [37]. None of the LFAs was reported for at-home testing, and none of them was capable of simultaneously measuring multiple cytokines on the same testing strip except for the LFA-CdTe QDs immunoassay [37] which measured one cytokine and one protein (the protein CRP).

### 3.4. Questionnaire-Based Methods as Supportive Health-Assessment Tools

Due to the immune system’s complexity, cytokines control the severity of health conditions through diverse pathways. Some diseases have heterogeneous impacts on cytokine levels. Thus, measuring cytokine levels solely is insufficient to perform a comprehensive assessment of the immune system functionality. An accurate medical history, family history, and physical examination are critical in developing the best immune status evaluation strategy. For example, the dysregulation of cytokine levels must be defined on age-appropriate reference ranges [56].

Cytokine production may trigger more than one disease at a time. These comorbidities contribute to the severity of each other.

For example, patients with Rheumatoid Arthritis (RA) and Systemic Lupus Erythematosus (SLE) show an increased insulin resistance (IR), which is linked to the systemic inflammation induced by certain inflammatory cytokines (TNF-α and IL-6) [57]. Severity of IR in patients with RA correlates with severity of inflammation (serum IL-6). Despite that both SLE and RA patients show similar serum TNF-α concentrations, IR in patients with SLE does not correlate with the severity of inflammation. The main contributor to IR in patients with SLE is obesity (high body mass index (BMI)) [58].

Therefore, we cannot determine the severity of IR by solely measuring the relevant cytokines. It is crucial to have additional information on other possible comorbidities, such as RA, lupus, and obesity. Another example is the patients with Knee Osteoarthritis. They express varying cytokine levels depending on the accompanying diseases, such as obesity, metabolic syndrome (MS), and type 2 diabetes mellitus (T2DM) [59]. In addition, the risk of developing ischemic heart disease (IHD) generally is associated with relatively low blood levels of Flt3 ligand, whereas, for patients suffering from abdominal obesity (AO), the relative risk of early ischemic heart disease (IHD) is linked to low levels of IL-4 [60].

Another factor that affects the immune system’s status is lifestyle [61]. Running a healthy lifestyle also plays a significant role in the immune system’s status. For example, a patient who suffers from heart disease but runs a healthy lifestyle exhibits different cytokine levels from another patient with the same disease but with unhealthy life habits. Therefore, it is essential to consider the health conditions, lifestyle, and other health-related aspects while assessing the immune system status.

In some cases, regular cytokine levels might be misleading. Cytokines may be affected heterogeneously by the diseases. For instance, MCP-1 correlates negatively with chronic active hepatitis B [62] but positively with autosomal dominant polycystic kidney disease (ADPKD) [63]. One expects a person with these two diseases to have contradicting effects on MCP-1, thus exhibiting a misleading regular level of MCP-1.

Therefore, the importance of employing health questionnaires emerges from the necessity of collecting information on all related health factors. Indeed, this importance is not confined to the conduction of a more reliable assessment of the immune system’s fitness. The detection of cytokines is an expensive procedure; thus, it needs to be reduced. However, the exclusion of cytokines that may be valuable to the assessment procedure is undesirable. In short, we must minimize the number of cytokines to be measured (for the interest of cost), and, at the same time, we must measure all the cytokines that may expose essential insights into the immune system.

As a result of this discussion, we conclude that the disturbed cytokines differ according to the diseases and comorbidities a person suffers. Hence, the cytokines involved in assessing the immune system fitness must be tailored to the person’s health condition to guarantee a reliable, inexpensive evaluation of the cytokine levels.

We collect eight health questionnaires. Four of the identified questionnaires are multiple-item questionnaires listed in Table 7, that include many items to assess multiple health aspects and the other four are single-item questionnaires listed in Table 8, and these consisted of 1-item to assess the self-rated/perceived health status. However, the self-rated/perceived health status was found to be strongly correlated with multiple health aspects; thus, the single-item questionnaires were used as a compressed tool to assess them. Essentially, these health factors are controlled by cytokines. In this study, we collect from literature the cytokines that are associated with each of these health factors. And in Section 5, we determine based on the impaired health factors and their associated cytokines the corresponding rapid PoC immunoassays retrieved earlier in this study.

The multiple-items questionnaires are listed and detailed across the three comparison factors (1) score range, (2) number of items, and (3) the major health factors inspected by the questionnaire in Table 7. Similarly, the single-item questionnaires are listed in Table 8.

#### 3.4.1. Multiple-Item Questionnaires

Multiple-item questionnaires aim at collecting information on different aspects of health. We study four questionnaires, one of them evaluated general health (GHQ) [19], the immune functioning assessment questionnaire (IFQ) that targeted immune-status related conditions [18], a reduced version of (IFQ) is the immune Status Questionnaire (ISQ) [20], and the last questionnaire is the immunodeficiency disease questionnaire (ISAQ) that focused particularly on infections and chronic immune diseases [17].

All the health questionnaires, except for the general health (GHQ) [19], asked the participants for an informed consent before filling the health questionnaire.

The four questionnaires were recorded on different scales. The score scales are shown in the 2nd column of Table 7. The largest scale (from 0 to 79) was utilized by the immune Status Questionnaire (ISQ) [20] (cf. last row, 2nd column). The general health questionnaire [19] utilized the smallest range from 0 to 28. Versprille et al. [18] incorporated two scales from 0 to 10 additional to the main scale. These two additional [0,10] scales were used independently of the main scale to capture the perceived immune functioning status.

GHQ comprised the highest number of items (28 items), whereas ISQ was the shortest questionnaire, with only 7 questions and two additional questions capturing the perceived immune functioning and general health. ISQ was intentionally designed to become a reduced version of IFQ.

The major health factors examined by each questionnaire are shown in the last column of Table 7. All the collected questionnaires investigated the physical aspects. Sport activities and lifestyle were investigated only by ISAQ. Besides the somatic aspects, GHQ examined a crucial aspect in the health status which was the psychological aspect reflected by the anxiety, insomnia, social dysfunction, and severe depression.

#### 3.4.2. Single-Item Self-Rated Health Questionnaires

A single-item questionnaire comprises one item question. Herein, we investigate the single-item questionnaires that are concerned with the perceived health status. Normally, the perceived health status questionnaires are built on the assumption that all health conditions directly impact the perceived health status. In other words, a poor perceived health status should be taken as an initial warning of a serious health issue. Subsequently, these health questionnaires can be used as a reliable assessment for various health aspects.

A significant correlation was discovered between the IFQ [24], the perceived immune functioning, the perceived health status, and the mental resilience, which was assessed using the BRS [64]. The perceived immune functioning and the perceived health status were evaluated using single-item questions on a scale from 0 to 10 (cf. the 3rd column in Table 8). In (Eriksson et al., 2001) [21], the global self-rated health status was evaluated using two different types of 1-item questions. One question asked about the current health status, and the other compared the health status with others of the same age and they were answered on two different scales from 1 to 5 and from 1 to 7, respectively. The largest scale from 0, indicating poor health to 100, indicating a better health status, on which the self-rated health was evaluated, was reported self-rated health questionnaire (SRH${}_{3}$) [23].

Only the self-rated health (SRH${}_{2}$) [22] and the Self-rated health (SRH${}_{3}$) [23] questionnaires investigated the need for hospitalization. All of the questionnaires except for the the self-rated health (SRH${}_{2}$) questionnaire [22] checked the mental health (cf. the 4th column in Table 8).

Only the perceived immune functioning and health questionnaire [24] asked the participants for an informed consent before filling the health questionnaire.

Furthermore, we observe that all questionnaires evaluated the health status at the present time, except for the self-rated health questionnaire (SRH${}_{3}$) [23], that considered the perceived health status during the past two weeks ‘two-weeks status’ (cf. 4th column, the second to last row in Table 8).

## 4. Discussion of the Findings on Rapid Technologies and Health Questionnaires

Throughout this work, we have been introduced to several technologies that contributed significantly to the great development in the design of the biosensors for cytokine measurements, where they delivered rapid results with small volumes of processed blood samples (e.g., plasma, serum, etc.). Essentially, all of the investigated assays confirmed an equivalent or superior performance to the standard ELISA. However, none of these assays could be used for at-home usage with no modifications. To open the horizon for assays to become convenient for at-home usage, the limits should be pushed a little bit further, and we need to add to the optimization of PoC platforms five essential features. The immunoassay that shows potentials for at-home usage should be characterized with five major properties:

1. multiple cytokine detection,
2. small volume samples,
3. smartphones-based readers,
4. rapid assay time, and
5. whole blood processing.

### 4.1. Multiple Cytokine Detection

As we have already emphasized that the immune system’s general health cannot be evaluated reliably by measuring only one cytokine level in the blood, hence, assays that can afford to measure multiple cytokines simultaneously carry a high value. Among all studied immunoassays, three of them could measure multiple cytokines simultaneously [26,27,30]. The 8-port manifold microfluidic biosensor [32] measured two cytokines with two proteins, and the sequentially-multiplexed biosensor [33] measured IL-6, as well as one protein, PCT.

Although none of the lateral flow assays produced multiple measurements, multiplexing has been investigated before in literature as it can be integrated easily to this type of immunoassays. The singleplexing in lateral flow assays can be extended to multiplexing with three different techniques [65]:

1. One-Strip xLFIA (Spatial Separation of Detection Sites) through increasing the number of test lines or dots on the same strip. Up to 32 measurements are feasible.
2. Array of Strips by placing multiple test strips separately on one holder.
3. Multiplexing LFA Based on the Probe by developing the detection element to capture multiple analytes at a time.

### 4.2. Small Volume Samples

We observe that three of the optical based assays (1 μL for the LSPR-AuNRs [26] and the LSPR-FACSNPs [27] immunoassays, and 5 μL for the PhloC immunoassay [28]), one of the electrochemical-based assays (5 μL for the 8-port manifold microfluidic biosensor [32]), and one of the colorimetic-based assays (2.5 μL for the plasmonic-based mobile biosensor [36]) consumed lower sample volumes with single-digit compared to 2 or 3-digits reported by the remaining ten immunoassays. In general, across all the immunoassays, except for the aptamer-based biosensor [31], the maximum sample volume required was 150 μL, which is equivalent to 3 drops of blood. Three drops of blood is relatively considered in the range of small sample volumes.

### 4.3. Smartphone-Based Readers

We find that most of the PoC biosensors required additional equipment for signal detection, such as potensatiosat or microscopy, making them suitable for PoC deployment only and not for at-home usage, except for the plasmonic-based mobile biosensor [36] and the LSPR-MoS${}_{2}$ immunoassay [29], that used a smartphone and an MoS${}_{2}$ photodetector as signal readers, respectively. In principle, LFAs delivered qualitative results, as in (hIL-8 XpressCard), unlike the LFA-AuNPs biosensor [5], the LFA-CdTe QDs biosensor [37], and the LFA (Milenia Biotec) IL-6 tests which used densitometry, portable fluorescence reader, and a camera, respectively, to convert the qualitative readouts to quantitative ones. However, the qualitative results delivered by the LFAs are no longer problematic because various smartphones-based solutions [66,67,68] were developed to produce quantitative results instead. Ruppert et al. [66] used an iPhone S5 with a simple dark box made from black cardboard and an open-source GNSplex R-package which included the Shiny app to compute concentrations from normalized or standardized intensities with a graphical user interface (GUI) to make the analysis of the image data easy.

Another smartphone-based LFA reader [67] was developed by Schneider et al. that was competitive with well-established LFA readers: QuickSensω100 (8sens. biognostic GmbH, Berlin, Germany, QuickSens) and the SkanSmart (Skannex AS, Oslo, Norway, SkanSmart). The LFA reader consisted of a microcontroller with a Bluetooth interface.

Unlike the former two solutions, Foysal et al. [68] proposed a solution that did not need any additional devices. They captured images to the LFA strip using the phone’s camera. Then, they trained a support vector machine classifier on defined quantities to classify measured quantities precisely.

### 4.4. Rapid Assay Time

This study is built principally on rapid assays with assay time less than 1 h. Two devices [27,31] delivered instant readouts in real-time with the former regulated the disturbed cytokine levels immediately by releasing a drug in living tissues. Four biosensors [26,32,33,35] reported a total assay time that ranged from 40 min to less than an hour, whereas the total assay time reported by the remaining nine assays ranged between 5 and 30 min. A total assay time of less than 30 min is considered rather short, thus, being convenient for at-home testing.

### 4.5. Whole Blood Processing

The ability to operate on whole blood samples (unprocessed samples) without the need to perform multiple preceding steps to convert the whole blood to serum or plasma is an essential feature for at-home testing. Firstly, we find that the majority of the identified immunoassays could not operate on unprocessed blood samples, except for two devices which are a device combined with a smartphone as a signal reader, the nanoparticle-based mobile biosensor [36], and a hybrid magneto-electrochemical miniaturized biosensor [35]. Indeed, we believe that adopting PoC assays to operate on whole blood samples is feasible, due to the recent advances in PoC testing which also promote rapid, easy solutions for plasma, and serum filtration from whole blood. Hauser et al. filtered 18 μL of plasma from 50 μL whole blood sample in less than 10 min with a fabricated device of dimensions: 80 × 20 mm [69]. Madadi et al. [70] extracted a tiny plasma sample 0.1 μL from 5 μL whole blood using a microfluidic-chip plasma separator within 5 min.

Another novel microfluidic blood filtration element was manufactured by Homsy et al. [71], which succeeded in extracting 12 μL of plasma from 100 μL of whole blood in less than 10 min.

## 5. Combination of Rapid Technologies with Health Questionnaires

As explained earlier, we collect from literature cytokines that are associated with each of the major health factors, as shown in the 3rd column in Table 9 and Table 10. In the 4th column, we align each of the health questionnaires with the appropriate rapid immunoassays that measure cytokines associated with the corresponding health factors.

Suppose we find that more than one immunoassay measures the corresponding cytokine. In that case, we select the immunoassay with the broader dynamic range, which measures/covers a higher number of the target cytokines or operates on whole unprocessed blood, because it is expected to have a greater diagnosis capacity.

From the 4th column in Table 9 and Table 10, we notice that most of the cytokines measured by the collected immunoassays associate with many questionnaires. Indeed, we believe that the immunoassays were implemented to measure cytokines that play a primary role in the health of the human body. These immunoassays had the broadest dynamic ranges and measured the highest number of cytokines among their peers that measured the same cytokines.

For example, IL-6 and IL-8 are found to be associated with all questionnaires. IL-10, an important cytokine, is found to associate with all the health questionnaires, except for the immunodeficiency disease questionnaire (ISAQ) [17]. Similarly, we notice that IFN-γ and TNF-α are linked to all the questionnaires but the perceived immune functioning and health questionnaire [24]. The cytokine immunoassays, [26,27,30,32,34,36], LFA (Milenia Biotec) and LFA (hIL-8 Xpress-Card), are linked to all the collected health questionnaires. Only two questionnaires are linked to IL-3, the immunodeficiency disease questionnaire (ISAQ) [17] and the immune functioning assessment questionnaire (IFQ) [20]; thus the hybrid magneto-based biosensor [35] is the only biosensor that measures IL-3 and is paired with them. IL-12p70 is associated with the self-rated health questionnaire (SRH${}_{1}$) [21] and the immunodeficiency disease questionnaire (ISAQ) [17] and is measured by the MCF immunoassay [30]. In addition, IL-1β is related mostly to skin problems in the chronic diseases in the self-rated health questionnaire (SRH${}_{2}$) [22], the immune Status questionnaire (ISQ) [18], and the immune functioning assessment questionnaire (IFQ) [20]. This cytokine is measured by the LSPR-MoS2 [29] and the MCF [30] immunoassays. The LSPR-FACSNPs immunoassay [27] is the only immunoassay (among the 15 immunoassays) that measured MCP-1 and TGF-β. MCP-1 is linked to all the health questionnaires, except for the perceived immune functioning and the health questionnaire [24] and the general health questionnaire (GHQ) [19], whereas TGF-β is linked to the self-rated health questionnaire (SRH${}_{2}$) [22], the self-rated health questionnaire (SRH${}_{3}$) [23], the immune Status questionnaire (ISQ) [18], and the general health questionnaire (GHQ) [19].

## 6. Data Mining

The ultimate goal of this work is to shed light on the existing potentials of implementing an efficient assessment tool of the immune system. After we have collected all the most advanced rapid immunoassays and the most informative health questionnaires, we must put these results in the context of real-world medical applications to promote public health. We need to build a model to interpret the information collected by the health questionnaire and the rapid cytokine immunoassay. This section presents a prototype model that integrates the health questionnaire and the rapid immunoassay to assess the fitness of a person’s immune system.

Up to this point, we have been concentrating on mechanisms for compiling essential data from health questionnaires and rapid immunoassays. However, determining whether these data reflect a un/fit immune system necessitates learning the definition of immune fitness beforehand. Therefore, we begin by discussing the definition of immune fitness. Then, we propose a solution to automatically derive this definition from literature, relying solely on machine learning techniques without human intervention. Finally, we discuss how to utilize this assessment system for assessing the immune system’s fitness.

### 6.1. Definition of Immune Fitness

Immune fitness is a general term that refers to the strength of the immune system. “Immune fitness can be defined as the capacity of the body to respond to health challenges (such as infections and/or fever) by activating an appropriate immune response in order to promote health and prevent and resolve disease, which is essential for improving quality of life” [111]. Various studies investigate how cytokine levels are disturbed by a particular disease in the presence of co-morbidities in different age groups, as already demonstrated in Section 3.4.

### 6.2. Derivation of the Immune Fitness Definition

Deriving a concrete definition of immune fitness might be a highly challenging task. In this subsection, we discuss how to derive an established definition of immune fitness from literature automatically using text mining techniques. Mainly, we aim at discovering how cytokines are associated with aging, diseases, and other health factors, such as physical and psychological aspects.

Topic modeling has been recently applied as an automated smart literature review in various domains. Asmussen et al. [112] proposed a framework that employed topic modeling for the exploratory literature review. Topic modeling has been used by Zhang et al. [113]. Specifically, they applied Latent Dirichlet Allocation (LDA) [114] for latent disease-gene knowledge discovery in biomedical literature. They managed to cover 17.8% of 146,245 disease-gene associations derived from 25 million PubMed articles across 159 topics. Trindade et al. [115] employed automatic text mining on 1590 articles on periodontitis and coronary heart disease to find that the molecules, C-reactive protein, IL-6, IL1-β, myeloperoxidase, and matrix metalloproteinase 9 are simultaneously associated with these diseases.

Similarly, we suggest using topic modeling to uncover associations between cytokines and the immune system and, thus, leverage the approach presented in the previous sections with new scientific discoveries about measuring specific cytokines in association with diseases and further co-morbidities.

**Figure 4:**
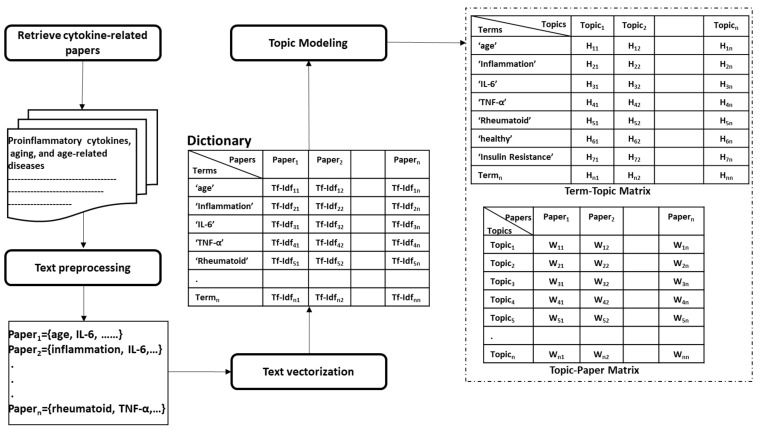
An illustrative example of the first four steps for modeling the topics expressed by the cytokines-related papers.

**Figure 5:**
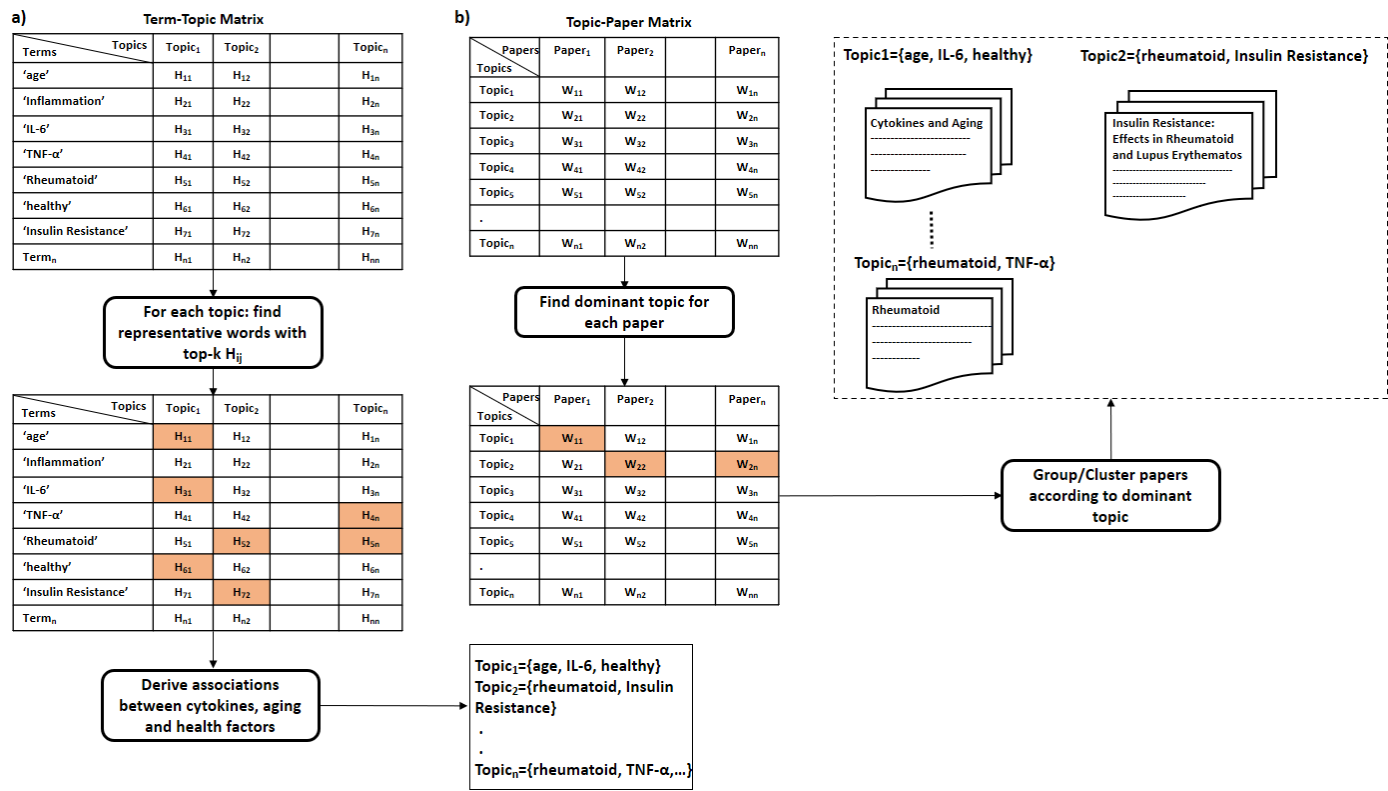
An illustrative example of steps (5 and 6): (**a**) describes the interpretation of topics; (**b**) demonstrates the clustering step for papers address similar topics.

**Figure 6:**
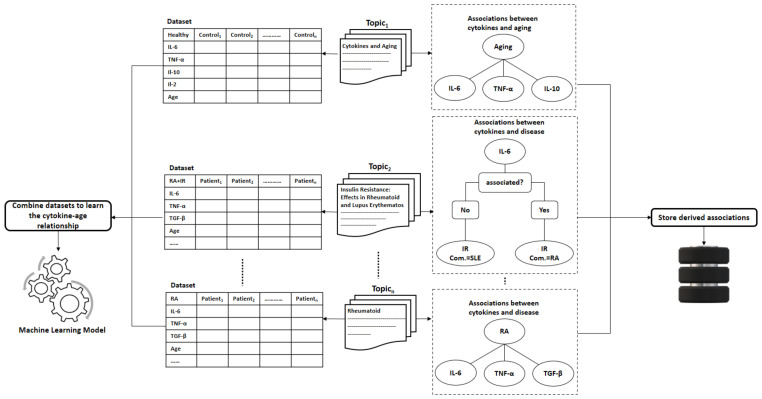
An example demonstrates collecting two types of data necessary for learning the immune fitness, the associations between cytokines and other health factors (e.g., disease), and the datasets containing cytokine measurements for RA patients, RA patients with IR, and healthy controls. The derived associations between cytokines, aging, and diseases are stored in the system, and the datasets are combined and fed to a machine learning model to learn the cytokine-age relationship. RA = Rheumatoid arthritis, IR = Insulin resistance.

For the topic modeling, we generally suggest following the same steps proposed by Asmussen et al. [112]. However, we replace LDA with NMF in our solution. There is no difference between the two methods, except that NMF does not generate normalized generated term-topic and document-topic matrices [116]. The topic modeling approach is described with the below-listed steps and summarized in Figure 4 (the first four steps) and Figure 5 (steps 5 and 6):

1. Collecting papers. We start with retrieving scientific papers that contain the term ’cytokine’ from digital libraries.
2. Text pre-processing: We extract keywords from papers by passing them into multiple pre-processing steps (tokenization, removing stop words, lemmatization, and stemming).
3. Text vectorization. Then we derive the term frequency-inverse document frequency (TF-IDF) features from collected papers. Afterwards, we build the paper-term matrix using the derived TF-IDF features.
4. Topic modeling. This is the main step of this procedure where the paper-term matrix comprising the TF-IDF features is passed to the topic modeling algorithm to reveal the underlying topics. One of the commonly used topic modeling techniques is the non-negative matrix factorization (NMF) [117]. NMF yields two matrices, the paper-topic matrix W and the topic-term matrix H. The number of topics to be derived is a crucial parameter that needs to be tuned prior to the topic modeling process. One way to find the optimal number of topics is to monitor the number of topics derived across several testing runs on randomly sub-sampled documents where the optimal number of topics should remain consistent across these testing runs. Moreover, one can evaluate the stability of the topics derived through tracking the fraction of documents pairs that are assigned to the same topic across the multiple testing runs [118].
5. Interpreting latent topics. This step implicitly derives associations between cytokines and other health factors/diseases. Topic modeling algorithms do not provide a straightforward interpretation of the derived topics, but we expect each topic to be presented by cytokines and their associated health factors. We can conclude the latent topics via finding the corresponding representative words for each topic. We consider the words with the top-k highest coefficients as the representative words for a topic, as shown in Figure 5a. The derived associations are stored in the system to be used later in the assessment procedure, as depicted in Figure 6. Even though we have attempted to derive these associations manually in Section 5, as shown in the 4th column in Table 9 and Table 10, this automatic method is more effective and practical.
6. Clustering of papers according to topic. We apply unsupervised clustering on topic-paper matrix, as illustrated in Figure 5b. Alternatively, we can assign each paper to the topic with the highest relevance score in W. Then, we group papers by the dominant topic. Semantic validation of the topics modeled should be performed by experts.
  Steps 5 and 6 are equivalent to the post-processing step proposed by Asmussen et al. [112]. As an output of this step, we expect that more than one disease to belong to the same topic in case of co-morbidities, such as Rheumatoid and insulin resistance.
  Various evaluation strategies can be followed to validate the topics modeled. One way is to validate them by experts semantically. Another way would be through evaluating the topics at the term level. Terms belonging to the same topic are supposed to be more similar to each other than to term in a different topic [113]. Therefore, the similarity scores between the two representative terms within the same topic should be smaller than the similarity scores to terms of a different topic. As each term is represented as a vector of coefficients in the term-topic matrix, we can use any similarity measure, such as cosine similarity or euclidean distance.
  Zhang et al. validated the derived disease-gene associations by topic modeling with annotated disease-gene associations by the Online Mendelian Inheritance in Man (OMIM) knowledge base [113]. Similarly, we can validate the proposed method by computing the overlapping proportion between the extracted disease/aging-cytokine associations and the manually derived associations in Section 5.
7. Collecting datasets. Now, for each group of scientific papers that belong to the same topic, we would collect, if available, datasets that contain the cytokine measurements for patients/controls.
8. Learning the Cytokines-Age relationship: Assuming terms that belong to the same topic are correlated, we can learn the correlations between diseases and cytokines. These correlations vary across different age slices. Therefore, researchers typically study these correlations with adjusting to age. To model the correlations between the cytokine levels and age, we can fit a machine learning model to estimate the age of the immune system $age_{immuneSystem}$ on datasets containing cytokine measurements collected from the scientific papers, as shown in Figure 6. The fitted machine learning models can be evaluated with cross-validation on the collected datasets.

### 6.3. Assessing the Person’s Immune System Fitness

**Figure 7:**
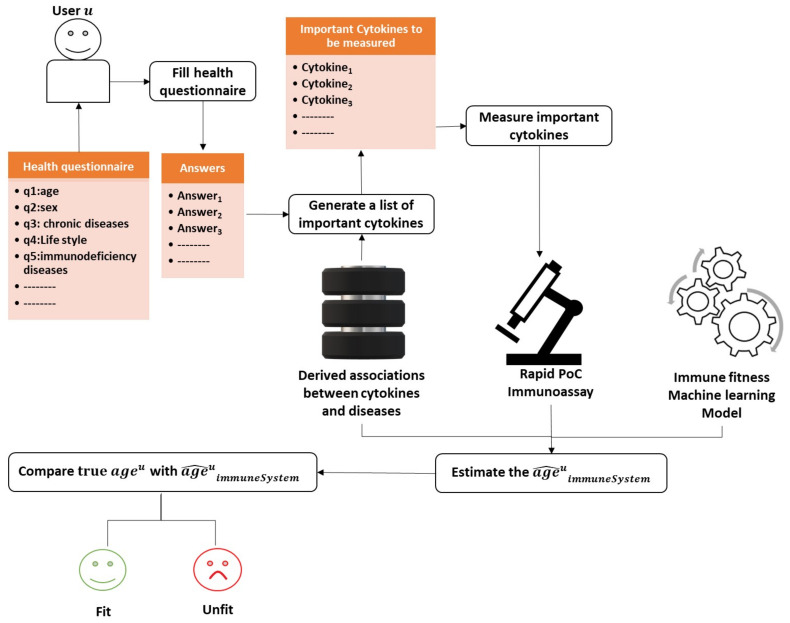
An abstract description of the usage of the assessment system tool. Users *u* answers the health questionnaire. Based on the user’s answers and from the previously derived associations between cytokines and diseases, the system generates a customized list of cytokines to be measured using a rapid PoC immunoassay. Then, the cytokine measurements are fed to the learned model to estimate the age of the age of the immune system. Lastly, the estimated age is compared with the real age to assess the immune fitness of the immune system.

Herein, we discuss a practical solution to assess the fitness of the immune system for a user. To this end, we follow a straightforward approach detailed in Figure 7. We employ the learned machine learning model to estimate the age of the immune system from the cytokine levels measured ideally using one of the rapid PoC immunoassays that are already retrieved in this study.

Say a user *u* aims at assessing the fitness of her/his immune system; the process is carried out through the following steps:

1. The user u starts with filling the health questionnaire.
2. As the disturbed cytokines change according to the diseases and the co-morbidities a user/person suffers from. The system is supposed to produce a customized list of important cytokines based on the user’s answers given to the health questionnaire and the previously derived associations from the method described in Section 6.2.
3. Then, the system measures only the cytokines identified as the most critical in the immune system with one of the powerful technologies mentioned previously in this study that requires less than a drop of blood (less than 50 μL). Reducing the number of measured cytokines is essential to promote efficiency and minimize the cost of the assessment procedure.
4. The age of the immune system is estimated by injecting the cytokine measurements into the learned machine learning model.
5. Let ${\hat{age^{u}}}_{immuneSystem}$ be the estimated age of the immune system of user *u*. We can compare ${\hat{age^{u}}}_{immuneSystem}$ with $age^{u}$ the real age of user *u* to decide whether the user’s immune system is fit or unfit.

## 7. Conclusions

We focused our research on rapid cytokine immunoassays with a total assay time of less than 1 hour in this work. We found that some of these immunoassays are suitable for point-of-care usage, and others could be with some slight development mature enough for home-usage. LFAs demonstrated their great potentials as PoC devices. Most of the immunoassays consumed less than three drops of blood. However, most of them could perform the measuring of a single cytokine only. Only two devices could process whole blood samples; thus, further optimization steps were required to develop devices capable of consuming whole blood samples. For a thorough evaluation of the immune system status, the cytokine levels must be studied in light of the patient’s health conditions or lifestyle. Therefore, it was necessary to also search for health questionnaires that collect information on several health aspects. We found questionnaires with multiple items and others with 1-item asking about the perceived immune status. Some of these questionnaires covered not only somatic health but also psychological health. Furthermore, we suggested a machine learning-based model that derives the definition of the immune system fitness and the associations between cytokines and other health factors automatically from literature. Finally, we propose an integrated solution that combines all the different components of this study to assess the person’s immune system fitness. In conclusion, we can say that there exist advanced technologies for measuring cytokine levels that, if paired with a health questionnaire, and boosted with machine learning methods, can hold promising potentials for the at-home assessment of immune system status to guarantee running a healthy life.

## Figures and Tables

**Table 1:** Juxtaposition of our features for the identified detection techniques to features from recent reviews on immunoassay readers for self-testing: although the reviews did not cover readers of cytokines, some of the features can be taken over.

Our Proposed Feature	Feature from Literature on Non-Cytokine Immunoassay Readers	Action
Assay time	Assessment speed	We used ‘Assay time’.
Sample volume and type	type and size of analyzed sample	We used ‘Sample’ as a composite feature.
Multiple simultaneous cytokine detection	n/a, since the reviews were not on cytokines	We used the feature ’Cytokines’.
Signal reader	support for smartphone/wearable, including readers that can be attached to smartphones or wearables	We stressed the use of smartphones whenever appropriate under the feature ‘Signal reader’.

**Table 2:** Rapid assays for cytokines measurement, Group O—*Optical* signal detection: mostly small sample volumes and low assay time make the techniques of this group attractive for PoC, but the need for specialized signal reading technology may be difficult to satisfy.

Optical Signal Detection									
**Technique**	**Sample**	**Cytokines**	**Dynamic Range (DR)**	**Limit of Detection (LoD)**	**Validity of Measurements**	**Assay Time**	**Signal Reader**	**Specific Disease**	**PoC ?**
LSPR-AuNRs [26]	1 μL of blood serum	IL-2 IL-4 IL-6 IL-10 TNF-α IFN-γ	Multiplex detection: 10–10,000 pg/mL	Multiplex detection: 6.46–20.56 pg/mL	Correlation with singleplex ELISA R${}^{2}$= 0.9726 Recovery within a range of 85–115%	40 min	dark-field imaging	Inflammatory responses after CBP surgery	×
LSPR-FACSNPs [27]	1 μL of biological sample	IL-6 MCP-1 TNF-α TGF-β	50–1000 pg/mL	18.96 pg/mL 14.57 pg/mL 32.62 pg/mL 22.08 pg/mL	Correlation with singleplex ELISA R${}^{2}$ = 0.9252	real time	dark-field microscopy	–	*√*
PhLoC [28]	5 μL of Lymphocytes separated from whole blood	IL-2	50–1000 pg/mL	50 pg/mL	–	15 min	UV-vis light, a microscope and a unit filter	–	*√*
LSPR-MoS2 [29]	Blood serum and plasma	IL-1β	$10^{6}$ pg/mL	250 fg/mL	–	10 min	Few-Layer MoS2 Photodetector	–	*√*
MCF [30]	150 μL of undiluted human serum	IL-6 IL-1β, IL- 12p70, TNF-α	1.5–2.0 log units	singleplex detection: 2.0–15.0 pg/mL Multiplex detection: 60–150 pg/mL	intra- and inter-assay coefficient of variation (CV) within 10%	20 min	HP ScanJet G4050 Scanner	–	*√*

**Table 3:** Rapid assays for cytokines measurements, Group O—*Optical* signal detection. Supporting information represented by the *dimensions* of the immunoassay, the place of *reaction*, and the **cost** of materials used.

Optical Signal Detection-Supporting Information			
**Technique**	**Dimensions**	**Reaction on**	**Cost**
LSPR-AuNRs [26]	An array of 480 stripe-shaped LSPR biosensing spots (25 μm × 200 μm)	AuNR microarray	–
LSPR-FACSNPs [27]	6 × sample-flow microfluidic channels (500 μm (W) × 2.5 cm (L) × 50 μm (H))	FACSNP microarray	–
PhLoC [28]	Measuring chamber (5 mm, 250 μm, and 200 μm)	the surface of the measuring chamber	–
LSPR-MoS${}_{2}$ [29]	SiO${}_{2}$ (300 nm thick) + few-layer MOS${}_{2}$ (0.65 nm thick) + SiO${}_{2}$(170 μm thick)	an SiO${}_{2}$ thin layer	–
MCF [30]	External dimensions of the MCF (4.5 ± 0.10 mm wide and 0.6 ± 0.05 mm thick)	the internal walls of the microcapillaries	–

**Table 4:** Rapid assays for cytokines measurements, Group E—*Electrochemical* signal detection: Immunoassays ask for larger sample volumes compared to immunoassays of optical detection. Due to the maturity of this detection technique, two biosensing tests are commercial products available in the market. Commercial tests are marked with ${}^{★}$.

Electrochemical Signal Detection									
**Technique**	**Sample**	**Cytokines**	**Dynamic Range (DR)**	**Limit of Detection (LoD)**	**Validity of Measurements**	**Assay Time**	**Signal Reader**	**Specific Disease**	**PoC ?**
aptamer-based biosensor [31]	in vitro 1 mL of WB	IFN-γ	10–500 pg/mL	10 pg/mL	–	vivo: real time vitro: 1 h	Shimadzu UV–Vis spectrophotometer model 2450	Inflam-mation	×
8-port manifold microfluidic biosensor [32]	5 μL of serum	IL-6 PF-4	Multiplex detection: sub pg/mL to well above ng/mL	Multiplex detection: 0.05–2 pg/mL	IL-6: correlation with singleplex ELISA R${}^{2}$ = 0.97418PF-4: correlation with singleplex ELISA R${}^{2}$ = 0.984	<1 h	CHI 1040A multi-potentiostat coupled with CHI 685 multiplexer	prostate cancer	×
sequentially-multiplexed biosensor [33]	150 μL of serum	IL-6	5–1000 pg/mL	5.0 pg/mL	–	40 min	ADC recordings via serial port communication	Inflam-mation	*√*
PoC-Proxim biosensor ${}^{★}$ [34]	100 μL of plasma or serum samples	IL-6	1–$10^{4}$ pg/mL	0.6 pg/mL	(n = 2) CV of concentration < 14% correlation with singleplex ELISA R${}^{2}$ = 0.96172	20 min	smartphone	Sepsis & cytokine release syndrome	*√*
hybrid magneto-based biosensor [35]	100 μL of WB, plasma, or serum samples	IL-3	∼10${}^{4}$ pg/mL	∼5 pg/mL	(n = 62) sensitivity = 91.3% specificity = 82.4%	≤1 h	smartphone	Sepsis & organ failure	*√*

**Table 5:** Rapid assays for cytokines measurements, Group E—*Electrochemical* signal detection. Supporting information represented by the dimensions of the immunoassay, the place of reaction, and the cost of materials used. ECBs = Electrochemical biosensors. Commercial tests are marked with ${}^{★}$.

Electrochemical Signal Detection-Supporting Information			
**Technique**	**Dimensions**	**Reaction on**	**Cost**
aptamer-based biosensor [31]	GC electrode (3 mm disks) + spectrophotometer (660 × 275 ×570 mm)	a glassy carbon (GC) rod	–
8-port manifold microfluidic biosensor [32]	Detection device (1 in. × 1 in. × 0.75 in.)	gold microelectrode	$0.50 in materials per 32-sensorcomplete system<$200
sequentially-multiplexed biosensor [33]	single-chip potentiostat (1.23 × 8.1 × 8.1 mm) + N ECBs	working electrodes (WE) for the ECBs	single-chip potentiostat (<20 USD)
PoC-Proxim technology ${}^{★}$ [34]	A handheld testing platform (11 × 4 × 3) cm${}^{3}$	ECBs in disposable Profile${}^{TM}$ cartridges	–
hybrid magneto-based biosensor [35]	Integrated device (10 × 1 × 2.5 cm${}^{3}$)	portable biosensor consisting of a disposable kit for blood processing	cost of goods ∼$50 for the device, and the reagent cost ∼$5 per test

**Table 6:** Rapid assays for cytokines measurements, Group C—*Colorimetric* signal detection: relatively larger samples are consumed compared to immunoassays based on optical detection. Signal readers are required to deliver quantitative readouts. None of the immunoassays measure multiple cytokines simultaneously. Only two LFAs are commercial product available in the market. Commercial products are marked with ${}^{★}$.

Colorimetric Signal Detection									
**Technique**	**Sample**	**Cytokines**	**Dynamic Range (DR)**	**Limit of Detection (LoD)**	**Validity of Measurements**	**Assay Time**	**Signal Reader**	**Specific Disease**	**PoC ?**
plasmonic-based mobile biosensor [36]	2.5 μL of WB	IL-6	variations in the basal concentration as small as 12.5 pg/mL	0.1 pg/mL	99% confidence	17 min	smartphone	sepsis	×
LFA-AuNPs [5]	150 μL of plasma	IL-6	1.25–9000 ng/mL	0.38 ng/mL	No statistical difference with cytometric bead array (CBA)n = 5, *t*-test (*p* < 0.05)	20 min	Canon T61 camera with 18–55 lens	visceral leishmania	*√*
LFA-CdTe QDs [37]	50 μL of serum	IL-6	1–1000 pg/mL	0.9 pg/mL	(n = 7) CV of concentration < 7% Errors < 14% correlation with singleplex ELISA R${}^{2}$ = 0.9994	30 min	portable fluorescence reader (PorFloR${}^{TM}$)	orthopedic implant-associated infections	*√*
LFA (Milenia Biotec GmbH, Germany) ${}^{★}$	100 μL of serum, plasma, cell culture supernatant, amniotic fluid	IL-6	10–10,000 pg/mL	10 pg/mL	–	20 min	densitometry (PicoScan) reader & chip reading card	–	*√*
LFA (hIL-8 XpressCard) ${}^{★}$	80 μL of biological fluids	IL-8	0.7–140 ng/mL	0.7 ng/mL	–	5 min	Naked eye	primary & secondary infections	*√*

**Table 7:** Multiple-item health questionnaires. (${}^{☆}$) indicates that an informed consent was obtained from the participants before filling the health questionnaire.

Questionnaire	Score Range	#Items	Major Health Factors
Immunodeficiency disease questionnaire (ISAQ) ${}^{★}$ (Peter et al., 2014) [17]	from 20 to 85	17	infections, immune diseases, sport activities, vaccination, life style, surgically removed immune organs radiation exposure, use of antibiotics, and immunosuppressants or immunostimulants
Immune Status Questionnaire (ISQ) ${}^{★}$ (Versprille et al., 2019) [18]	from 7 to 35	7	sudden high fever, diarrhea, headache, skin problems, muscle and joint pain, and common cold and coughing
	from 0 to 10	1-item question	perceived immune functioning
	from 0 to 10	1-item question	perceived general health
General health questionnaire (GHQ) (Goldberg et al., 1979) [19]	from 0 to 28	28	somatic symptoms, anxiety, insomnia, social dysfunction, and severe depression
Immune functioning assessment questionnaire (IFQ) ${}^{★}$ (Reed et al., 2015) [20]	from 0 to 79	19	common cold, influenza, cold sores pneumonia, sepsis, and skin infections

**Table 8:** Single-item health questionnaire for the perceived health status. (${}^{★}$) indicates that an informed consent was obtained from the participants before filling the health questionnaire.

Questionnaire	Question	Score Range	Found to be Strongly Correlated with
Self-rated health questionnaire (SRH${}_{1}$)(Eriksson et al., 2001) [21]	“How would you rate your health status?”	from 1 to 5	mental health, chronic diseases,
	“How would you rate your health status?”	from 1 to 7	health care visits, chronic functional limitations,
	“How would you rate your health status compared to that of others of your own age?”	from 1 to 5	lifestyle factors, psycho-social factors, and quality of life.
Self-rated health questionnaire (SRH${}_{2}$) (Cislaghi et al., 2019) [22]	“How is your health in general?”	from 1 to 5	chronic health conditions, chronic functional limitations, 28 diagnosed health conditions, demand of hospitalization, consultation of medical, or surgical specialist and medicine use
Self-rated health questionnaire (SRH${}_{3}$) (Meng et al., 2014) [23]	“Which can best represent your health today?”	from 0 to 100	diagnosed diseases, physical functional status, two-weeks status, mental health status, and the need for hospitalization
Perceived immune functioning and health questionnaire ${}^{★}$ (Lantman et al., 2017) [24]	–	from 0 to 10	mental resilience BRS and IFQ

**Table 9:** Multiple-item health questionnaires paired with the identified immunoassays. Each of the health factors is linked to a set of cytokines that are found to have a role in health conditions related to the corresponding health factor. In the last column, we list the immunoassays that can be employed to measure the associated cytokines. SLE = Systemic lupus erythematosus, OA = Osteoarthritis, AD = Atopic Dermatitis, and RA = Rheumatoid arthritis.

Questionnaire	Major Health Factors	Found to be Linked To	
		Cytokines	Immunoassays
Immunodeficiency disease questionnaire (ISAQ) (Peter et al., 2014) [17]	infections	bacterial infections: IL-2, IL-6, TNF-α [72] HIV: IL-2, (IFN)-γ, IL-4, IL-10, IL-6, IL-8, TNF-α [73] common cold: IL-1β [74], MCP-1 [75] SARS-CoV-2: IL-3 [76]	{IL-6}: [32,34,36] and LFA (Milenia Biotec) {IL-6, IL-1β, IL-12p70, TNF-α}: [30] {IL-3}: [35]
	immune diseases	RA, SLE, Psoriasis: IL-2, IL-4, IL-8, MCP-1 [77] Diabetes: IL-6, IL-18, TNF-α [78] Systemic Sclerosis: PF-4 [79] Crohn’s disease: IL-12p70 [80]	{IL-8}: LFA (hIL-8 XpressCard) {IL-1β}: [29] {PF-4, IL-6}: [32] {MCP-1}: [27] {IL-2, IL-4, IL-6, TNF-α, IFN-γ}: [26]
	sport activities	IL-6, TNF-α, IFN-γ, IL-2 [81]	
Immune Status Questionnaire (ISQ) (Versprille et al., 2019) [18]	sudden high fever	IL-6, IL-1β [82]	
	diarrhea	IL-6, TNF-α [83]	{IL-4, IL-6, IL-10, TNF-α
	headache	IL-1β [84]	IFNγ}: [26]
	skin problems	AD, Psoriasis: IFNγ [85,86] IL-4 [87] IL-1β [88]	{IL-6, MCP-1, TNF-α, TGF-β}: [27] {IL-1β}: [29] {IL-6}: [32,34,36], LFA (Milenia Biotec)
	muscle and joint pain	OA: IL-6, IL-10 [89] RA:TNF-α, IL-6, TGF-β [90]	{IL-8}: LFA (hIL-8 XpressCard) {IL-6, IL-1β, TNF-α }: [30]
	common cold and coughing	IL-1β, IL-6, IL-8, TNF-α [74] MCP-1 [75]	
General health questionnaire (GHQ) (Goldberg et al., 1979) [19]	somatic symptoms	TNF-α, IFN-γ [91]	{IL-2, IL-4, IL-6, IL-10, TNF-α, IFN-γ}: [26]
	anxiety	IL-6, TNF-α, IL-10 [92]	{IL-6, TNF-α, TGF-β}: [27]
	insomnia	IL-2, IL-6, IL-8, TGF-β, IL-4 [93]	{IL-8}: LFA (hIL-8 XpressCard)
	social dysfunction, and severe depression	IL-6, IL-10, TNF-α, IFN-γ [94]	{IL-6}: [32,34,36], LFA (Milenia Biotec) {IL-6, TNF-α}: [30]
Immune functioning questionnaire (IFQ) (Reed et al., 2015) [20]	cold, influenza, and cold sores	IL-1β, IL-6, TNF-α [74], MCP-1 [75]	{IL-4, IL-6, IL-8, IL-10, TNF-α, IFN-γ}: [26]
	pneumonia	IL-6, IL-8 and IFN-γ, IL-10 [95]	{IL-1β}: [29]
	sepsis	IL-1β, IL-6, IL-7, IL-8, IL-10, IFN-γ, MCP-1 and TNF-α [96] IL-3 [35]	{IL-6, IL-1β, TNF-α}: [30] {IL-6, MCP-1, TNF-α}: [27] {IL-3}: [35]
	skin infections	IFN-γ [85,86] IL-4 [87] IL-1β [88]	{IL-8}: LFA (hIL-8 XpressCard) {IL-6}:[32,34,36] LFA (Milenia Biotec)

**Table 10:** Single-item health questionnaire for the perceived health status paired with the identified immunoassays. Each of the health factors is linked to a set of cytokines that are found to have a role in health conditions related to the corresponding health factor. In the last column, we list the immunoassays that can be employed to measure the associated cytokines. SLE = Systemic lupus erythematosus, OA = Osteoarthritis, and RA = Rheumatoid arthritis.

Questionnaire	Major Health Factors of the	Found to be Linked To	
	Validation Questionnaire	Cytokines	Immunoassays
		Diabetes: IL-6, TNF-α [78]	{IL-2, IL-4, IL-6, IL-10, TNF-α,
	chronic diseases	Crohn’s disease: IL-12p70 [80]	IFN-γ}: [26]
Self-rated health (SRH)		RA, SLE, Psoriasis: IL-2, IL-4, IL-8,	{IL-6}: [32,34,36], LFA (Milenia Biotec)
(Eriksson et al., 2001) [21]		MCP-1 [77]	{IL-8}: LFA (hIL-8 XpressCard)
	psycho-social factors	Depression: IL-6, IL-10, TNF-α, IFN-γ [94]	{IL-6, IL-12p70, TNF-α}: [30]
		Anxiety: IL-6, TNF-α, IL-10 [92]	{IL-6, MCP-1, TNF-α}: [27]
		Diabetes: IL-6, TNF-α [78]	
	chronic health conditions	IL-1β [97]	
		RA, SLE, Psoriasis: IL-2, IL-4, IL-8,
		MCP-1 [77]	
	chronic functional	OA: IL-6, IL-10 [89]	{IL-2, IL-4, IL-6, IL-10, TNF-α,
	limitations	RA: TNF-α, IL-6, TGF-β [90]	IFN-γ}: [26]
		Asthma: IL-4 [98]	{IL-6}: [32,34,36], LFA (Milenia Biotec)
		Hypertension: IL-6, IL-8, TGF-β,	{IL-8}: LFA (hIL-8 XpressCard)
Self-rated health (SRH)		TNF-α [99]	{IL-6, IL-1β, TNF-α}: [30]
(Cislaghi et al., 2019) [22]		HPV: L-1β, IL-2, IL-4, IL-8, IL-10, TNF-α,	{IL-6, MCP-1, TNF-α, TGF-β}: [27]
		TGF-β [100]	{IL-1β}: [29]
	28 diagnosed health	Parkinson: TNF-α, IL-1β, IL-2, IL-4, IL-6,	
	conditions	TGF-β [101]	
		Lumbar disc: IFN-γ [102]	
		chronic bronchitis: TNF-α, IL-1β, MCP-1, IL-6, IL-8, TGF-β [103]	
		Tumor: IL-1β, IL-4 and IL-6, IL-2 [104]	
		Alzheimer: TNF-α, TGF-β, IFN-γ [105]	
		Angina: IL-10 [106]	
		cirrhosis of liver: TGF-β, IL-1β, IL-10,	
		TNF-α, IFN-γ [107]	
		Diabetes: IL-6, TNF-α [78]	
		acute coronary syndromes: PF-4 [108]	{IL-2, IL-4, IL-6
	chronic diseases	RA, SLE, Psoriasis: IL-8, MCP-1 [77]	IL-10, TNF-α, IFN-γ}: [26]
Self-rated health (SRH)		HIV: IL-2, IFN-γ, IL-4, IL-10	{IL-6}: [30,34,36], LFA (Milenia Biotec)
(Meng et al., 2014) [23]		IL-6, IL-8, TNF-α [73]	{IL-8}: LFA (hIL-8 XpressCard)
	physical functional status	OA: IL-6, IL-10 [89]	{IL-6, MCP-1, TNF-α, TGF-β}: [27]
		RA: TNF-α and IL-6, TGF-β [90]	{IL-6, PF-4}: [32]
	mental health status	Schizophrenia: IL-2, IL-6, IL-8,	
		IL-10 [109,110]	
Perceived immune	mental resilience	Schizophrenia: IL-2, IL-6,	{IL-2, IL-6, IL-10}: [26]
functioning and health		IL-8, IL-10 [109,110]	{IL-8}: LFA (hIL-8 XpressCard)
(Lantman et al., 2017) [24]			{IL-6 }: [27,30,32,34,36],
			LFA (Milenia Biotec)
